# Two-eyed seeing: Embracing the power of Indigenous knowledge for a healthy and sustainable Ocean

**DOI:** 10.1371/journal.pbio.3001876

**Published:** 2022-10-21

**Authors:** Kelsey Leonard, Pier Luigi Buttigieg, Maui Hudson, Kenneth Paul, Jay Pearlman, S. Kim Juniper

**Affiliations:** 1 School of Environment, Resources, and Sustainability, Faculty of Environment, University of Waterloo, Ontario, Canada; 2 Helmholtz Metadata Collaboration, GEOMAR, Kiel, Germany; 3 Te Kotahi Research Institute, University of Waikato, Hamilton, Aotearoa, New Zealand; 4 Wolastoqey Nation, New Brunswick, Canada; 5 IEEE, Paris, France; 6 FourBridges, Port Angeles, Washington, United States of America; 7 Ocean Networks Canada, University of Victoria, Victoria, British Columbia, Canada; Smithsonian Institution, UNITED STATES

## Abstract

Indigenous knowledge is often disregarded and opportunities for positive change are lost. The authors of this Perspective argue that, to protect the Ocean, Indigenous and western knowledge systems need to be combined in a way known as "two-eyed seeing".

## Teaser

Indigenous knowledge is often disregarded and opportunities for positive change are lost. To protect the Ocean, a “two-eyed seeing” approach combining Indigenous and western knowledge systems can create advancements while empowering coastal Indigenous Peoples.

Two heads are better than one. So they say when it comes to finding solutions to problems: bringing more perspectives to the table increases the likelihood of producing impactful and meaningful ideas. The same can be said when it comes to protecting the Ocean. Research data produced by sensors and laboratory analyses are invaluable, but so is the Indigenous marine knowledge acquired today and through millennia of experience and observation.

“Two-eyed seeing”—*Etuaptmumk* in Mi’kmaw—is a concept championed by Mi’kmaq Elder Albert Marshall. It proposes that integrating the diverse scientific traditions, systems, and methods of Indigenous and western knowledge can result in innovative advancements [1]. When it comes to Ocean sustainability, Indigenous ways of knowing, such as two-eyed seeing, are critical in environments that Indigenous People have stewarded for millennia.

With this in mind, there is an urgent need for Ocean policy affecting Indigenous Peoples’ lands and waters to be Indigenous led and informed from full and respectful partnerships between Indigenous coastal communities and their western scientific counterparts. Recognizing Indigenous ways and perspectives, such as the two-eyed seeing Mi’kmaq concept, provides a pathway for further empowering coastal Indigenous Peoples and correcting the marginalization of their knowledge systems, experience, and scientific practice. We arrived at this consensus during the panel session on Ocean Observation and Indigenous Knowledge at POGO-22, the annual general meeting of the Partnership for the Observation of the Global Ocean, which took place virtually in January 2021. The discussion also carried into POGO-23, which took place virtually in January 2022.

Over the years, the relationship between western scientists and Indigenous Peoples has been tumultuous, demanding, and, typically, colonial. Some of the Indigenous panelists at POGO-22 stated that many non-Indigenous scientists are unaware of Indigenous Ocean knowledge and its relevance to Ocean sustainability challenges. For example, the belief that Indigenous knowledge is static, would lead to the incorrect assumption that Indigenous Peoples would not know about the effects of climate change (Box 1). Even when engagement does occur, western science often “extracts” Indigenous knowledge as if it is a resource for the taking. Ocean research not grounded in community and which lacks Indigenous consent and leadership will lead to intergenerational harm and broken trust. For example, mapping a coastline or ocean may have great effects for protecting a nation’s security, boosting trade or developing natural resource industries, but it might also result in more vessel traffic, which, in turn, increases the risks of oil spills, marine debris, and loss of marine mammals. Even creating a census of marine life—vital to biodiversity conservation or tracking the effects of climate change—can easily be incomplete if it ignores Indigenous knowledge, and potentially harmful if it results in uniform regulatory controls on Indigenous Peoples’ access to ceremonial, cultural, and subsistence fisheries. Indigenous seafood harvesting and stewardship are vital focal points for cultural knowledge transmission and the sustainability of the food source itself; therefore, reduced access to marine resources can have drastic consequences on all aspects of life, including for the maintenance of Indigenous languages, cultural practices, and economic independence.

**Box 1. An invaluable source too often ignored**

One of the richest sources of information that Indigenous Peoples bring to knowledge-pairing partnerships are the direct, year-round observations made by people out on the land and on the sea, over many generations. At POGO-22, panelist Austin Ahmusuk provided examples from coastal Alaska.

Hunters, mariners, trappers and dog mushers in his region of Alaska have been noticing signs of climate warming since the 1950s: in ocean currents, in ice conditions and snowfall, and in the colonization of their peninsula by shrubs and trees. However, their warnings found few listeners outside of their region —until decades later, when “the rest of the world took notice that severe climate change was occurring,” he said.

Even then, researchers seeking to compile Indigenous knowledge in communities and the region would ignore direct observations by individuals. They wanted statistically robust generalities, not “anecdotal” evidence. What they missed was that important, climate-relevant traditional knowledge exists at the family level, thanks to direct observations made by “folks like myself,” said Ahmusuk, “who spend a great deal of time hunting and fishing.”

Even though direct observations have a low level of experimental control, they offer the highest possible degree of ecological validity, especially in situations where the physical outcomes of ongoing climate change processes are readily visible. Observers are uniquely qualified to capture detail, identify anomalies, and interpret what they have seen.

“There is a race to the Arctic to understand change … how it impacts communities,” Ahmusuk said. “Unfortunately, though, Arctic communities are not always involved in how the research is designed, how it’s planned or how that research can benefit our communities.”

Since 2007, the United Nations Declaration on the Rights of Indigenous Peoples [2] has recognized that respect for Indigenous knowledge, cultures, and traditional practices contributes to sustainable and equitable protection of the environment. When it comes to Ocean research, this respect can and should be implemented in everyday practice. Atlantic First Nations across what is currently known as Canada have been instrumental in advancing two-eyed seeing through the Atlantic Policy Congress of First Nations Chiefs Secretariat and the Unama’ki Institute of Natural Resources, both of which work to protect Indigenous Ocean knowledge and advance cross cultural exchanges with western scientists for collaborative fisheries science and management [3]. Additional best practices exist for mobilizing two-eyed seeing for Ocean health [4,5,6] and were identified in a 2021 successful partnership review [7]. Aha Honua—the Coastal Indigenous Peoples’ Declaration developed during the 2019 OceanObs’19 conference in Hawai’i [8]—called upon the global Ocean-observing community to implement “meaningful” research partnerships with coastal Indigenous Peoples. We all need to recognize the value of Indigenous knowledge, science, and experience in shaping our global understanding of the Ocean.

Ocean sustainability is vitally important at the local level, and there is an urgent need for research outcomes that directly benefit Indigenous nations and communities. Coastal Indigenous communities must be respected as key Ocean rightsholders and decision-makers. Indigenous leadership in Ocean research and policy is vital to supporting Indigenous culture, ceremony, and food sovereignty. Moving forward will require an open, two-eyed seeing mind. We need to create best practices—alongside nurturing on-the-ground relationships in Indigenous communities—to build bridges and create true partnerships between Indigenous Peoples, Indigenous communities, Ocean scientists, and Ocean-observing institutions.

When designing an Ocean research, observation, or monitoring project, scientists must engage Indigenous communities at an early stage to cocreate project goals and methods. Questions can be as simple as: “I have an interest in XYZ; do you have a mutual interest in this?”; “How do we work together to make sure it is a reciprocal relationship?”; and, of equal importance, “How does this benefit the Ocean?” Vitally, research funding itself should reinforce these values, requiring and resourcing genuine cocreation with both western science/environmental management and Indigenous coastal communities while supporting all in identifying and advancing each community’s own research. This can empower Indigenous Peoples to focus on their priorities and be in a better position to choose their research partners.

Two-eyed seeing is a skill that would benefit the entire Ocean community. Indigenous Ocean ethics are rooted in marinescapes across the planet, and increasing their adoption can be accelerated by documenting wise practices that employ them well. During our panel discussion, representatives of the Intergovernmental Oceanographic Commission of UNESCO’s Ocean Best Practices System (OBPS) committed to increasing the archiving and visibility of Indigenous ethical frameworks for Ocean observing. Early work is based on input from Indigenous communities in Greenland, Svalbard, Siberia, and Alaska; however, the OBPS welcomes communities across all regions.

Overall, we underscore the need to concretely implement Indigenous Ocean ethics, such as two-eyed seeing, as a means to address the underrepresentation of coastal Indigenous Peoples, their wisdom, and their experience in Ocean sustainability research, decisions, policies, and actions. As the global community undertakes the work of the UN Decade of Ocean Science for Sustainable Development (2021–2030), the time to act is now.
